# Sebaceous Carcinoma of the Penis: A Rare, Dangerous Clinical Entity and the Importance of Immunohistochemistry in Diagnosis

**DOI:** 10.1155/2023/6944296

**Published:** 2023-01-27

**Authors:** Vikram Sahni, David S. Cassarino

**Affiliations:** ^1^Drexel University College of Medicine, Philadelphia, USA; ^2^Kaiser Foundation Hospital, Department of Pathology, Los Angeles, USA

## Abstract

We report a very rare case of pathologically confirmed sebaceous carcinoma of the glans penis with multiple areas of lymphovascular and perineural invasion and multiple lymph node metastases. The importance of immunohistochemical staining in diagnosis is also reviewed.

## 1. Introduction

Sebaceous carcinoma (SC) is a rare tumor that is a potentially aggressive malignant cutaneous neoplasm arising from adnexal sebaceous glands [1]. While most commonly a malignancy of the head and neck, especially the eyelids and periorbital area, isolated cases have been reported throughout the body, including the shoulder, trunk, and genitals [2]. Pathologic correlation and immunohistochemistry can aid in the diagnosis and differentiation of SC from other conditions given its nonspecific presentation as an enlarging, firm nodule [2].

## 2. Case Report

**Figure 1:**
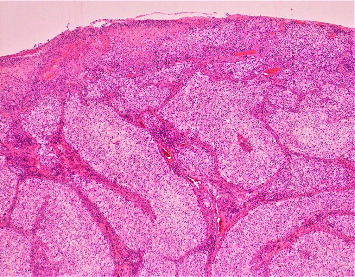
SC at low power (4x magnification) shows a large multinodular tumor invading the dermis with diffuse overlying ulceration present.

**Figure 2:**
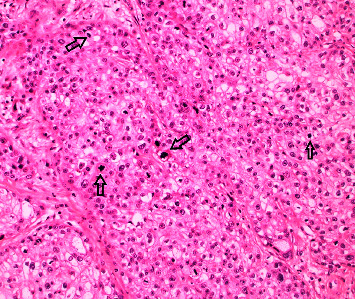
SC at high power (20x magnification) shows marked cytologic atypia, abundant clear-staining cytoplasm, and numerous mitotic figures.

**Figure 3:**
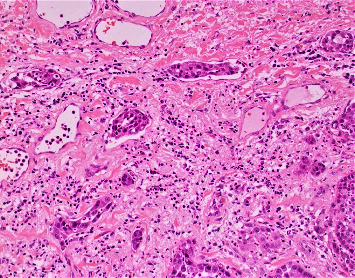
SC with lymphatic invasion.

The patient was a 78-year-old man with a history of diabetes mellitus, hypertension, moderately differentiated prostatic adenocarcinoma with no evidence of metastasis, and a nodular BCC who presented for evaluation of a lesion on his glans penis. It was described as a 2 × 2 cm irregular, friable mass. A deep shave biopsy of the lesion revealed nearly complete epidermal ulceration with replacement by an in-situ and diffusely infiltrative, atypical-appearing clear cell tumor (Figure 1). Within the epidermis, the cells displayed diffuse pagetoid spread. Large irregular nests and lobules of atypical-appearing clear cells with abundant multivacuolated cytoplasm, nuclear indentations, and vesicular to hyperchromatic-staining nuclei made up the majority of the tumor (Figure 2). Given the multivacuolated cytoplasm and nuclear indentations, the tumor was highly suspicious for sebaceous carcinoma. Included in the initial differential diagnosis were a high-grade clear-cell SCC and a metastatic clear-cell carcinoma. Lymphovascular invasion was also present, involving multiple small vessels (Figure 3).

Immunohistochemical staining allowed for further classification of the tumor. A cytokeratin (CK7) stain was strongly and diffusely positive, consistent with its usual expression in sebaceous carcinoma. The epithelial membrane antigen (EMA) stain also showed moderate to strong positivity in most of the cells, with accentuation and highlighting of the cytoplasmic vacuolations. An androgen receptor (AR) was also positive, with diffuse nuclear staining. In addition, p63 and C5/6 stains were positive, with strong nuclear staining for p63 and peripheral weak staining for C5/6; this is consistent with a primary cutaneous tumor. Prostate-specific antigen (PSA) and prostatic acid phosphatase (PAP) immunohistochemical stains were also performed, given the patient's history of prostatic carcinoma, and were negative. A GATA-binding protein 3 (GATA3) stain was also negative.

The patient had a wide excision (resection of the glans and distal shaft of the penis) with clear margins, but extensive lymphovascular invasion was present. Core needle biopsies of the right ilioinguinal, right external iliac, right superficial and deep inguinal, the apex of the femoral triangle, and posterior/lateral right external iliac vein lymph nodes were carried out for appropriate staging, and all lymph nodes revealed a poorly differentiated carcinoma with focal involvement of the extranodal soft tissue, consistent with metastatic SC. Immunohistochemical stains on the lymph node samples were strongly and diffusely positive for CK7 and EMA, similar to the findings in the penile lesion. Skin nodules from the patient's right leg also revealed poorly differentiated metastatic SC and exhibited retained expression of mismatch repair proteins (MMRPs), including MLH1, MSH2, MSH6, and PMS2.

The patient was offered palliative chemotherapy, but given his poor health status and metastatic disease involving multiple lymph nodes, the skin, and potentially the brain (based on imaging studies), chemotherapy was not pursued. The patient expired 9 months later.

## 3. Discussion

Sebaceous carcinoma of the penis is a poorly characterized entity with less than 10 verified cases in the literature. Whereas SC of the eyelids displays a low rate of lymph node involvement and distant metastasis, penile sebaceous carcinoma tends to be metastatic at presentation [3]. It is necessary to consider SC in the differential diagnosis for penile masses, despite SCC making up 95% of cases [3].

The etiology of SC remains unclear, but the majority of cases arise from de novo mutations. Sebaceous carcinoma associated with Muir-Torre syndrome displays loss of MMRP gene expression and microsatellite instability [4]. With its origin in sebaceous cells, the upper eyelid is the most often affected area, making up 28% of eyelid malignancies in the Asian population [1].

Immunohistochemical stains can aid in the diagnosis of sebaceous carcinoma (SC) and differentiation from basal cell carcinoma (BCC) and squamous cell carcinoma (SCC). In a study exploring immunohistochemistry in SC, quantification of immunopositivity of cells was documented using a 4-tier system (0% was considered to be negative, 1–25% (1+), 25%–75% (2+), and >75% (3+)) [5]. 27 cases of SC were studied using a broad panel of IHC markers, including EMA, CK7, Ber-EP4, Factor XIIIa, AR, p53, adipophilin, PGRMC1, SQS, and ABHD5. EMA was expressed in all 27 cases, with diffuse staining (3+) in 21 cases, and CK7 was expressed in 89% of cases, with diffuse 3+ staining in 21 cases. Adipophilin, PGRMC, SQS, and ABHD5 were expressed in varying amounts (100%, 81%, 52%, and 70%, respectively). EMA was negative in all cases of BCC, and CK7 was positive in 29%. EMA was expressed in 16 cases (72% of SCC) and CK7 in 2 cases (9%). All cases of SCC and BCC were negative for adipophilin, PGRMC1, SQS, and ABHD5, demonstrating that these are potential novel markers for the differentiation of SC, with adipophilin being the most sensitive [5]. Other studies found similar outcomes with EMA, Ber-Ep4, and ADP utility in the differentiation of SC from BCC and SCC, respectively [6]. N-cadherin can also be used as a potential biomarker in diagnosing SCC [7].

Treatment options for SC of the penis include local excision, partial penile amputation, and radical penile amputation [3]. For lymph node metastasis and extranodal involvement, a protocol of paclitaxel and carboplatin, used for orbital SC, may be warranted [3, 8].

Our case emphasizes the importance of recognizing that SC can present in unusual sites and should be considered in the differential diagnosis of malignant tumors, even in atypical locations such as the penis. Histologic features of this case, such as multivacuolated cytoplasm and vesicular to hyperchromatic-staining nuclei, are important clues to the diagnosis [9]. Lymph node metastasis is common at presentation with SC of the penis, illustrating the importance of keeping it in the differential diagnosis of penile masses [8].

## Data Availability

No data were used to support this study.
